# Macroscopic and microscopic evaluation of flapless alveolar perforations on experimental tooth movement

**DOI:** 10.1590/2177-6709.23.6.073-079.oar

**Published:** 2018

**Authors:** Jose Luis Munoz Pedraza, Mariana Marquezan, Lincoln Issamu Nojima, Matilde da Cunha Gonçalves Nojima

**Affiliations:** 1 Private practice (Rio de Janeiro/RJ, Brazil).; 2 Universidade Federal de Santa Maria, Departamento de Estomatologia, Disciplina de Ortodontia (Santa Maria/RS, Brazil).; 3 Universidade Federal do Rio de Janeiro, Faculdade de Odontologia, Departamento de Ortodontia e Odontopediatria (Rio de Janeiro/RJ, Brazil).; 4 Case Western Reserve University, Department of Orthodontics (Cleveland, USA).

**Keywords:** Tooth movement techniques, Orthodontics, Bone remodeling.

## Abstract

**Objective:**

The aim of this study was to evaluate a flapless surgical technique as an alternative to traditional alveolar corticotomy used to accelerate orthodontic tooth movement (OTM).

**Methods:**

To induce OTM in Wistar rats, 40 cN of orthodontic force were applied to the maxillary left first molars. Forty rats were distributed into control groups (CG1, CG3, CG7 and CG14) and experimental groups (*n*= 5), in which alveolar perforations were made using a spear-shaped guide bur (EG1, EG3, EG7, EG14). Euthanasia dates were set at 1, 3, 7 and 14 days, respectively, after tooth movement began. The amount of OTM was measured with a caliper, and osteoclasts present in the periodontal ligament of the mesial root of the moved tooth were counted by means of histological evaluation (tartrate-resistant acid phosphatase staining, TRAP).

**Results:**

Although there was no difference in the amount of OTM within subgroups of corresponding experimental periods (*p*> 0.05), when EG14 and CG14 were compared, a larger number of osteoclasts was counted in the experimental group (*p*< 0.00).

**Conclusion:**

The authors concluded that flapless cortical alveolar perforations led to more intense osteoclastic activity on the fourteenth day; nevertheless, no evidence of accelerated OTM could be noted.

## Resumo

**Objetivo:**

o objetivo do presente estudo foi avaliar a técnica cirúrgica sem retalho como alternativa à tradicional corticotomia alveolar utilizada para acelerar o movimento dentário experimental.

**Métodos:**

para induzir a movimentação dentária experimental em ratos Wistar, foram aplicados 40 cN de força ortodôntica aos primeiros molares superiores esquerdos. Quarenta ratos foram distribuídos nos grupos (n = 5) controles (GC1, GC3, GC7 e GC14) e experimentais (GE1, GE3, GE7, GE14), nos quais foram realizadas perfurações alveolares com uma ponta lança. As datas de eutanásia foram estabelecidas em 1, 3, 7 e 14 dias, respectivamente, após o início do movimento dentário. A quantidade de deslocamento dentário foi medida com um paquímetro e os osteoclastos presentes no ligamento periodontal da raiz mesial do dente movimentado foram contados por meio de avaliação histológica (coloração por fosfatase ácida resistente ao tartarato, TRAP).

**Resultados:**

embora não tenha havido diferença na quantidade de deslocamento dentário dentro dos subgrupos dos períodos experimentais correspondentes (*p*> 0,05), quando GC14 e GE14 foram comparados, um número maior de osteoclastos foi contado no grupo experimental (*p*< 0,00).

**Conclusão:**

os autores concluíram que as perfurações alveolares corticais sem retalho levaram a uma atividade osteoclástica mais intensa no décimo quarto dia; entretanto, nenhuma evidência de movimento dentário acelerado pôde ser notada.

## INTRODUCTION

In some clinical situations, orthodontic tooth movement (OTM) can be a complex task due to the severity of malocclusions, or to diminish the biologic responses commonly seen in periodontally compromised adults and patients with general health problems.^1^ ^,^ ^2^ Therefore there is demand for therapeutic approaches that facilitate OTM whenever a factor involving difficulty is associated, thus reducing the time of active orthodontic treatment. Selective alveolar bone corticotomy before beginning with orthodontic treatment is outstanding among the available alternatives,^3^ ^-^ ^9^ because increased local tissue metabolism in response to surgical trauma can accelerate OTM.^5^ ^,^ ^10^

According to Köle,^11^ alveolar corticotomy is a surgical procedure in which incisions limited to the bone cortex are made. For treating severe dentoskeletal discrepancies, the author suggested a clinical combination of interdental cuts and subapical osteotomies, followed by use of a removable orthodontic appliance. Later, the surgical approach was restricted to alveolar corticotomy in association with orthodontic therapy, shortening treatment duration.^12^ Ever since it was first reported, the success of alveolar corticotomy was related to outlining the bone blocks connected by cancellous bone only, thereby offering less resistance to orthodontic forces.^11^

The combination of orthodontic movement with selective alveolar corticotomy was addressed again in 2001.^13^ According to the authors, the increased efficiency of orthodontic treatment did not result from facilitated bone block movement, outlined by the corticotomy, but from an increment in bone metabolism. This concept was described as the Regional Acceleratory Phenomenon (RAP).^13^ Change in bone physiology would result in a local reduction of trabecular bone density, offering less resistance to moving the desired teeth. It has been demonstrated, in a systematic review, a reduction of the time varying between 28 and 66% for accomplishment of the orthodontic movement.^14^

In spite of the success of accelerated OTM, the aggressive nature of traditional corticotomy resulted in reluctance by both patients and the dental community to proceed with this technique.^15^ To overcome the disadvantages of corticotomy, less invasive and flapless techniques were developed, such as corticision^10^ ^,^ ^16^ and micro-osteoperforations.^17^ In corticision, a reinforced scalpel is used as a thin chisel to separate the interproximal cortices transmucosally without reflecting a flap.^10^ ^,^ ^16^ In micro-osteoperforations, a disposable device with cutting edge designed for this purpose (Propel Orthodontics, Ossining, NY) can be used.^17^ Kits for miniscrew placement often have tips for cortical perforation that could be used as an alternative to this device. The aim of this study was to evaluate the macroscopic and microscopic effects of a flapless cortical alveolar perforation technique, using a spear-shaped guide bur from a miniscrew placement kit, during OTM. 

## MATERIAL AND METHODS

### Experimental model

This *in vivo* study used 40 male Wistar rats (*Rattus norvegicus*), weighing 250-280 g and approximately 90 days old. Animals were kept in a vivarium of the *Universidade Federal do Rio de Janeiro* (UFRJ), under ideal conditions throughout the experiment. The research project had been previously approved by the Ethics Committee for Animal Research at the Health Sciences Center of UFRJ.

Animals were randomly distributed into control (CG) and experimental groups (EG), split into 4 subgroups of 5 animals each, namely CG1, CG3, CG7 and CG14; EG1, EG3, EG7 and EG14, according to the date of euthanasia set at 1, 3, 7 and 14 days, respectively, after tooth movement began. In the experimental subgroup animals, the maxillary left first molar was moved in a mesial direction and flapless perforations were made on the buccal and palatal cortical plates, limited by the adjacent alveolar process. Control group animals were submitted exclusively to orthodontic movement, following the same protocol described for the experimental groups.

Animals were sedated via intraperitoneal injection of 1ml/Kg anesthetic solution, made up of equal parts of 100 mg/ml ketamine hydrochloride and 20 mg/ml xylazine hydrochloride, for placement of the orthodontic device. 

**Figure 1:**
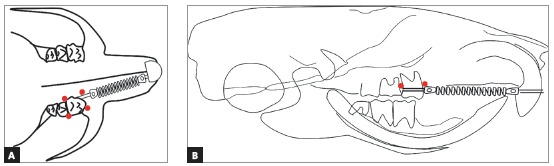
Schematic drawing of the orthodontic coil spring and perforation points (circles) in occlusal (A) and lateral (B) views.

A 7-mm NiTi closed coil spring (Dental Morelli Ltda; Sorocaba, São Paulo, Brazil) stretched up to the maxillary incisors (Fig 1) applied a force of 40 cN to the first molar, measured with a tension gauge (Dentaurum, Ispringen, Baden-Württemberg, Germany). The mandibular incisors were trimmed for the purpose of preserving the integrity of orthodontic devices. 

**Figure 2:**
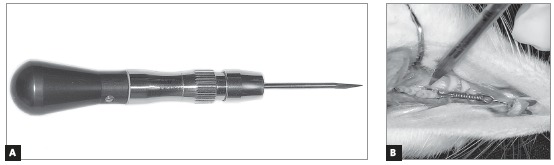
Spear-shaped guide bur (FML 70) (A) used to perform perforations in attached gingiva (B).

Once the devices were placed, two cortical alveolar perforations were made on the buccal plate and another two on the palatal plate of all animals in EG1, EG3, EG7 and EG14. Perforations were made in the attached gingiva of mesial and distal portions of the maxillary left first molar (Fig 1), using a spear-shaped guide bur (FML 70, S.I.N., Sistema de Implante Nacional Ltd., São Paulo, Brazil), attached to a miniscrew manual driver (CDM 02, S.I.N., Sistema de Implante Nacional Ltd., São Paulo, Brasil) (Fig 2). No flaps were raised during this procedure. Perforations of all groups were performed only at baseline.

### Quantification of OTM

The amount of OTM was measured with an orthodontic caliper (Odin, Orthopli, Philadelphia, Pennsylvania, USA), positioned from a point marked on the composite covering the maxillary incisors to the cervical portion of the mesial surface of the maxillary left first molar. Distances were measured immediately after placement of the devices and before euthanasia of each animal with an overdose of anesthetic solution composed of ketamine hydrochloride and xylazine hydrochloride. The amount of OTM was calculated by subtracting the final from initial distances. All measurements were repeated twice by the same examiner, and a mean value was obtained and registered.

### Histological analysis

**Figure 3:**
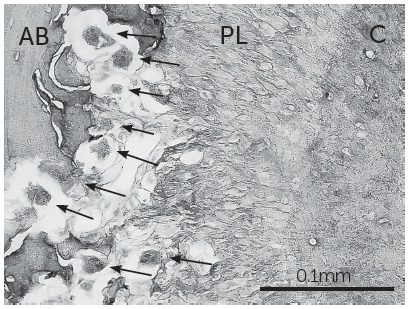
Micrograph of the mesial root of a first molar and its supporting tissue processed for TRAP histochemistry to identify osteoclast cells (arrows) (400x magnification). AB = alveolar bone; PL = periodontal ligament; C = cementum; bar: 0.1 mm.

The maxillary bones were dissected and the anatomical blocks were fixed in 4% formaldehyde buffered solution. Bone structures were demineralized using Allkimia^®^ (Campinas, São Paulo, Brazil) and were subsequently embedded in paraffin blocks (Paraplast, Sigma-Aldrich Co, St. Louis, MO, USA). Cross-sections 5-µm thick were obtained from the cervical third of the root, placed on glass slides, deparaffinized, hydrated in water, and then stained. Tartrate-resistant acid phosphatase (TRAP) histochemistry stain (TRAP kit n. 387, Sigma Chemicals, Saint Louis, Missouri, USA) was used to count the number of osteoclasts on the adjacent alveolar bone surface throughout the entire extension of the periodontal ligament of the mesial root of the maxillary left first molar, and to estimate the degree of bone resorption. Cells were considered osteoclasts if they were multinucleated, TRAP positive (red-brownish color), and located on or close to bone surfaces (Fig 3). Counting was done manually under light microscopy (microscope Nikon Eclipse E600, 400x magnification). 

### Statistical analysis

Statistical Package for the Social Science software (version 17, SPSS Inc., Chicago, Illinois, USA) was used for analyzing the results. Reproducibility of the osteoclast count measurements was tested by the Intraclass Correlation Coefficient (ICC = 0.981), and 30% of total sample measurements were repeated after a 15-day interval. Data were submitted to descriptive statistical analysis. Normality and homogeneity of variables were assessed by Shapiro-Wilk and Levene tests, respectively, to a level of significance of 0.05. Since normal and homogenous distribution were verified, the Analysis of Variance (ANOVA) and Tukey multiple comparisons tests were used to evaluate intergroup differences. The level of significance adopted was of 0.05.

## RESULTS

The amount of OTM showed no significant differences between control and experimental subgroups after the same periods of activation. In the control group, a difference was detected between the first and fourteenth day of orthodontic movement (*p*= 0.001) (Table 1).

**Table 1:** Mean and standard deviation (SD) of amount of OTM and number of osteoclasts and results of ANOVA/Tukey tests.

	OTM (mm)	Number of osteoclasts
CG1	0.41 ± 0.10^a^	27.60 ± 8.20^a,b^
CG3	0.76 ± 0.05^a,b,c^	37.60 ± 10.03^a,b^
CG7	0.76 ± 0.10^a,b,c^	47.80 ± 17.56^a,b^
CG14	1.1 ± 0.30^c^	50.60 ± 15.46^b^
EG1	0.67 ± 0.25^a,b^	12.60 ± 3.91^a^
EG3	0.73 ± 0.28^a,b^	62.00 ± 28.15^b^
EG7	0.68 ± 0.07^a,b^	48.40 ± 16.68^b^
EG14	0.78 ± 0.07^b,c^	107.20 ± 24.38^c^
	P=0.001	P=0.000

Different letters indicate statistical difference at α=0.05% (ANOVA/Tukey).

Each column refers to an independent statistical test.

Histological analysis demonstrated a significant difference in the number of osteoclasts between control and experimental subgroups on the fourteenth day after tooth movement began, with increased quantities of osteoclasts in EG14 (*p*< 0.000) (Table 1). 

## DISCUSSION

OTM is a process of bone remodeling in response to mechanical loading. The velocity of OTM is influenced by bone turnover, bone density, and the degree of hyalinization of the periodontal ligament in response to the forces applied.^10^ The elimination of cortical resistance or increased local tissue metabolism might prevent excessive pressure buildup in the periodontal ligament and subsequent hyalinization.^8^ In this context, surgical techniques performed in alveolar bone can accelerate OTM by increasing bone turnover and reducing hyalinization in the periodontal ligament.^10^

Ever since Local Alveolar Corticotomy (LAC) was first used by Heinrich Köle^11^ (in 1959) to facilitate orthodontic movement, new surgical interventions have been proposed to decrease the considerable risks to the periodontium and maintaining vitality of the associated teeth.^18^ The aim was to attain better treatment results in a reduced period of time; therefore, with this focus, less invasive and extensive variations of the original surgical technique have proved effective in achieving more complex tooth movements.^4^ ^,^ ^19^ The association between local alveolar corticotomy and lyophilized bone grafts has been described as a method to shorten conventional orthodontic treatment time,^13^ ^,^ ^15^ ^,^ ^20^ together with other minimally invasive methods, such as smaller cortical incisions, either associated or not with piezosurgery. However, these approaches have been described in the literature mainly as case reports.^4^ ^,^ ^12^ ^,^ ^16^ ^,^ ^19^ ^,^ ^21^ ^,^ ^22^ Thus, the biological responses derived from rapid OTM have not been fully understood. Thus, the aim of this experimental study was to evaluate the amount of OTM and periodontal reactions that resulted from a flapless surgical technique involving cortical alveolar perforation, considered less invasive from a biological standpoint. The experimental periods were chosen with the purpose of contemplating the three phases of orthodontic movement: dental movement within the periodontal ligament (1 to 3 days), lag phase (7 days), and frontal bone resorption (14 days).^23^

The results showed no statistically significant difference in the amount of OTM between the experimental and control subgroups. This result was in agreement with a previous experimental study that evaluated the effect of two distinct magnitudes of force applied with and without corticision on the rate of OTM in rats. Corticision was unable to induce clinical changes after two weeks of OTM, irrespective of the force magnitude.^10^ On the other hand, this result disagreed with a previous clinical study using flapless cortical microperforations.^17^ The site of perforations must have influenced the result. While we performed four perforations - two on buccal and two on lingual side - all in cervical third of the roots, Alikhani et al^17^ performed three perforations along the root length on the buccal surface. The purpose of the choice to perform the perforations on the buccal and lingual surfaces of the bone tissue was to increase bone turnover, based on conventional corticotomy techniques, in which cuts were performed buccally and lingually.^24^ ^,^ ^25^ However, it seemed to be important to perform perforations along the root length to obtain better results.

Specifically on the fourteenth day of OTM (although there was no statistical difference) there was an important numerical difference in the rate of OTM between the control and experimental groups. The control group obtained 0.32 mm more OTM. Studies with larger samples could better clarify this issue. 

The TRAP enzyme is an osteoclast marker that can be used to quantify them in different tissues. Changes in bone resorption are usually associated with changes in the osteoclast count, suggesting that TRAP could be a useful marker for bone resorption.^26^ Multinucleated TRAP-positive cells adhered or close to bone were considered osteoclasts and therefore counted, throughout the periodontal ligament. The present results showed a gradual progression in the number of osteoclasts in the control subgroups during the experiment. However when analyzing the effect of alveolar perforations, an expressive response with a statistically significant increase in osteoclast counts was verified over two specific time intervals during the experiment, from the first (EG1) and seventh (EG7) days, to the third (EG3) and fourteenth (EG14) days of orthodontic movement. Intergroup analysis also showed evidence of a significant difference in osteoclast counts on the fourteenth day of tooth movement, with higher numbers in the experimental subgroup (EG14) than in control subgroup (CG14), suggesting that the tissue responded favorably to the surgical procedure performed in this study. Previous studies using surgical techniques to accelerate OTM in rats showed little differences in their results. While Wang et al^27^ found an increase in osteoclast number on the third day after corticotomy, Murphy et al^10^ found no difference in osteoclast number and activity after 14 days of tooth movement in rats, despite of corticision being performed or not. These results must be explained by methodological differences, including differences in surgical techniques. 

The strength of the present study lies in accomplishing the evaluation of an innovative technique in research, previously cited only in clinical cases. Research in animals has greatly contributed to knowledge in the field of etiology, prevention and treatment of oral diseases. There are advantages to using animals, such as the possibility of better local control, less genetic diversity when using litters, ease of sample collection, higher number of replications and ease of microscopic analysis.^28^ However, some biological differences should be considered between humans and animals, such as the faster metabolism of rats. Therefore, clinical results in our patients could be expected in a longer period of time, compared to those found in experimental studies. Moreover, animal studies are at the bottom of the pyramid of scientific evidence and their findings should be extrapolated into clinical practice with caution.

As science is a cumulative process, this paper significantly improves the knowledge base beyond what is already published on the topic through histological findings. However, further researches with similar methodology as presented in this study and longer observation periods are necessary in order to verify whether the significant difference in osteoclast count found between groups on the fourteenth day of the experiment will continue to evolve and result in a clinically significant difference in amount of OTM. Moreover, the authors suggest that further studies performing perforations along the entire root length should be conducted to verify the histological reactions; in addition, a positive control group should be used, in which traditional corticotomy (the gold standard technique) should be performed. 

## CONCLUSIONS

It could be concluded that flapless cortical alveolar perforations led to more intense osteoclastic activity on the fourteenth day of tooth movement (verified microscopically); nevertheless, no evidence of accelerated OTM could be noted macroscopically.

## References

[1] Yamaguchi K, Nanda RS (1992). Blood flow changes in gingival tissues due to the displacement of teeth. Angle Orthod.

[2] Machado CC, Nojima Mda C, Rodrigues e Silva PM, Mandarim-de-Lacerda CA (2012). Histomorphometric study of the periodontal ligament in the initial period of orthodontic movement in Wistar rats with induced allergic asthma. Am J Orthod Dentofacial Orthop.

[3] Anholm JM, Crites DA, Hoff R, Rathbun WE (1986). Corticotomy-facilitated orthodontics. CDA J.

[4] Hwang HS, Lee KH (2001). Intrusion of overerupted molars by corticotomy and magnets. Am J Orthod Dentofacial Orthop.

[5] Bhattacharya P, Bhattacharya H, Anjum A (2014). Assessment of corticotomy facilitated tooth movement and changes in alveolar bone thickness - A CT Scan Study. J Clin Diagn Res.

[6] Gkantidis N, Mistakidis I, Kouskoura T, Pandis N (2014). Effectiveness of non-conventional methods for accelerated orthodontic tooth movement a systematic review and meta-analysis. J Dent.

[7] Hassan AH, Al-Saeed SH, Al-Maghlouth BA, Bahammam MA, Linjawi AI, El-Bialy TH (2015). Corticotomy-assisted orthodontic treatment A systematic review of the biological basis and clinical effectiveness. Saudi Med J.

[8] Hoogeveen EJ, Jansma J, Ren Y (2014). Surgically facilitated orthodontic treatment a systematic review. Am J Orthod Dentofacial Orthop.

[9] Liem AM, Hoogeveen EJ, Jansma J, Ren Y (2015). Surgically facilitated experimental movement of teeth: systematic review. Br J Oral Maxillofac Surg.

[10] Murphy CA, Chandhoke T, Kalajzic Z, Flynn R, Utreja A, Wadhwa S (2014). Effect of corticision and different force magnitudes on orthodontic tooth movement in a rat model. Am J Orthod Dentofacial Orthop.

[11] Kole H (1959). Surgical operations on the alveolar ridge to correct occlusal abnormalities. Oral Surg Oral Med Oral Pathol.

[12] Generson RM, Porter JM, Zell A, Stratigos GT (1978). Combined surgical and orthodontic management of anterior open bite using corticotomy. J Oral Surg.

[13] Wilcko WM, Wilcko T, Bouquot JE, Ferguson DJ (2001). Rapid orthodontics with alveolar reshaping two case reports of decrowding. Int J Periodontics Restorative Dent.

[14] Hoogeveen EJ, Jansma J, Ren Y (2014). Surgically facilitated orthodontic treatment a systematic review. Am J Orthod Dentofacial Orthop.

[15] Cassetta M, Pandolfi S, Giansanti M (2015). Minimally invasive corticotomy in orthodontics a new technique using a CAD/CAM surgical template. Int J Oral Maxillofac Surg.

[16] Kim SJ, Park YG, Kang SG (2009). Effects of Corticision on paradental remodeling in orthodontic tooth movement. Angle Orthod.

[17] Alikhani M, Raptis M, Zoldan B, Sangsuwon C, Lee YB, Alyami B (2013). Effect of micro-osteoperforations on the rate of tooth movement. Am J Orthod Dentofacial Orthop.

[18] Bell WH, Levy BM (1972). Revascularization and bone healing after maxillary corticotomies. J Oral Surg.

[19] Chung KR, Oh MY, Ko SJ (2001). Corticotomy-assisted orthodontics. J Clin Orthod.

[20] Wilcko WM, Ferguson DJ, Bouquot JE, Wilcko MT (2003). Rapid orthodontic decrowding with alveolar augmentation case report. World J Orthod.

[21] Germec D, Giray B, Kocadereli I, Enacar A (2006). Lower incisor retraction with a modified corticotomy. Angle Orthod.

[22] Sebaoun JD, Surmenian J, Dibart S (2011). [Accelerated orthodontic treatment with piezocision: a mini-invasive alternative to conventional corticotomies]. Orthod Fr.

[23] Reitan K (1960). Tissue behavior during orthodontic tooth movement. Am J Orthod Dentofacial Orthop.

[24] Shoreibah EA, Salama AE, Attia MS, Abu-Seida SM (2012). Corticotomy-facilitated orthodontics in adults using a further modified technique. J Int Acad Periodontol.

[25] Wilcko MT, Wilcko WM, Pulver JJ, Bissada NF, Bouquot JE (2009). Accelerated osteogenic orthodontics technique a 1-stage surgically facilitated rapid orthodontic technique with alveolar augmentation. J Oral Maxillofac Surg.

[26] Halleen JM, Tiitinen SL, Ylipahkala H, Fagerlund KM, Vaananen HK (2006). Tartrate-resistant acid phosphatase 5b (TRACP 5b) as a marker of bone resorption. Clin Lab.

[27] Wang L, Lee W, Lei DL, Liu YP, Yamashita DD, Yen SL (2009). Tisssue responses in corticotomy- and osteotomy-assisted tooth movements in rats: histology and immunostaining. Am J Orthod Dentofacial Orthop.

[28] Valladares JV, Souza JB, Estrela C (2001). Ética em Pesquisa. Metodologia Científica.

